# Activity of *Fusarium oxysporum*-Based Silver Nanoparticles on *Candida* spp. Oral Isolates

**DOI:** 10.3390/nano12030501

**Published:** 2022-01-31

**Authors:** Maísa Santos Fonseca, Daniela Méria Rodrigues, Ana Rita Sokolonski, Danijela Stanisic, Luiz Marcelo Tomé, Aristóteles Góes-Neto, Vasco Azevedo, Roberto Meyer, Danilo Barral Araújo, Ljubica Tasic, Ricardo Dias Portela

**Affiliations:** 1 Laboratório de Imunologia e Biologia Molecular, Instituto de Ciências da Saúde, Universidade Federal da Bahia (UFBA), Salvador 40110-100, BA, Brazil; maisa.s.fonseca@gmail.com (M.S.F.); danmeria@gmail.com (D.M.R.); rmeyer@ufba.br (R.M.); 2 Laboratório de Bioquímica Oral, Instituto de Ciências da Saúde, Universidade Federal da Bahia (UFBA), Salvador 40110-100, BA, Brazil; anasokolonski@gmail.com (A.R.S.); danilobarral81@hotmail.com (D.B.A.); 3 Laboratório de Química Biológica, Instituto de Química, Universidade Estadual de Campinas (UNICAMP), Campinas 13083-970, SP, Brazil; stanisicdanijela@yahoo.com (D.S.); ljubica@iqm.unicamp.br (L.T.); 4 Laboratório de Biologia Molecular e Computacional de Fungos, Instituto de Ciências Biológicas, Universidade Federal de Minas Gerais (UFMG), Belo Horizonte 31270-901, MG, Brazil; lmrtome@ufmg.br (L.M.T.); arigoesneto@icb.ufmg.br (A.G.-N.); 5 Laboratório de Genética Celular e Molecular, Instituto de Ciências Biológicas, Universidade Federal de Minas Gerais (UFMG), Belo Horizonte 31270-901, MG, Brazil; vasco@icb.ufmg.br

**Keywords:** biogenic silver nanoparticles, *Candida albicans*, *Candida dubliniensis*, fungicidal drugs, prosthetic stomatitis

## Abstract

*Candida* spp. resistant to commercially available antifungals are often isolated from patients with oral candidiasis, a situation that points to the need for the development of new therapies. Thus, we evaluated the activity of *Fusarium oxysporum*-based silver nanoparticles (AgNPs) on *Candida* spp. isolated from denture stomatitis lesions. *Candida* isolates were molecularly identified and submitted to susceptibility assays using AgNPs and commercial fungicides. The interference on biofilm formation and the mechanisms of action of AgNPs on *Candida* spp. were also investigated. Scanning electron microscopy was used to evaluate the morphology of AgNP-treated *Candida*. *Candida albicans* was the most frequent species isolated from denture stomatitis cases. All *Candida* spp. were susceptible to AgNPs at low concentrations, except *Candida parapsilosis*. AgNPs caused surface damage, cell disruption, and biofilm formation inhibition. The ergosterol supplementation protected *C. albicans* against the AgNP action. AgNPs are effective against *Candida* spp. and can be faced as a promising new therapeutic agent against oral candidiasis.

## 1. Introduction

Denture stomatitis is an infection of the oral cavity characterized by inflammation and erythema, and predisposing conditions increase the ability of opportunistic *Candida* fungi to cause oral candidiasis [1]. This infection is caused by *Candida albicans* and by other non-*Candida albicans* (NCA) species, such as *Candida parapsilosis*, *Candida tropicalis* and *Candida glabrata* [2].

Infections caused by *Candida* spp. are extensively recurrent [3], being this recurrence, as well as the development of chronic infections, associated with the formation of biofilm [4]. *Candida* species are competent biofilm-forming microorganisms, and these structures are correlated with an enhanced resistance to antifungals [5]. The inappropriate use of antifungals has resulted in the emergence of multidrug-resistant fungi [6]. Moreover, less attention has been given to the development of new antifungals [7]. The use of alternative technologies, such as metallic nanoparticles, has been considered in the last few years since they present significant antimicrobial and antiviral properties [8]. Among these nanoparticles, silver nanoparticles (AgNPs) are the most studied nanocompounds as a consequence of their several applications [9].

AgNPs consist of nanostructures originated from silver that can be synthesized by chemical or biogenic methods [10,11]. The chemical synthesis of metallic nanoparticles is used for large-scale production; however, a significant amount of energy is needed, and some toxic reagents that can negatively affect human health and the environment are used in this synthesis protocol [10]. In contrast, biogenic synthesis uses microorganisms [12], and does not require toxic reagents, being considered as an ecofriendly alternative [11]. In dentistry, silver nanoparticles synthesized by several chemical processes have been used as antimicrobials and in dentistry material development [13]. These nanoparticles have been successfully synthetized on a poly (methyl methacrylate) resin (acrylic glass), which led to a significant reduction on the adhesion and viability of *C. albicans* [14]. The diversity of AgNPs studies on dentistry can be applied to the clinical field if rigorous criteria are included on their evaluation [15]. 

AgNPs exhibit a broad-spectrum antimicrobial activity against bacteria and fungi [16]. Biogenic AgNPs are a good option among nanoparticles with antimicrobial activities since they present low production cost and good efficacy, stability, and durability [17,18]. Green synthesis using spruce bark extracts generated biogenic AgNPs that inhibited the growth and biofilm formation by several Candida species [19]. Furthermore, AgNPs synthetized using *Terminalia catappa* leaf extract exhibited an inhibition of the biofilm formation by multidrug-resistant *Pseudomonas aeruginosa*, methicillin-resistant *Staphylococcus aureus* and *Candida albicans* [20]. Additional AgNP mechanisms of action include an increase in the production of reactive oxygen species and changes in the general cellular metabolism and fluidity of the target cell plasma membrane [21,22]. 

Considering the potential of biogenic AgNPs as antimicrobial agents, we aimed to evaluate their antifungal activity against *Candida* spp. isolated from patients with prosthetic stomatitis. A comparative evaluation of the efficiency of commercial antifungals and AgNPs against planktonic *Candida* spp. and their associated biofilm was also performed. Additionally, an evaluation of the effects of AgNP on *Candida* specimens was performed.

## 2. Materials and Methods

### 2.1. Fungal Strains, Media and Commercial Antifungals 

The *Candida* spp. reference strains used in this study were kindly provided by Fundação Oswaldo Cruz (FIOCRUZ, Rio de Janeiro, Brazil) and included four *C. albicans* (IOC 2508, IOC 2517, IOC 3703, and IOC 3704), *C. parapsilosis* (CP310), *C. tropicalis* (CT 309), and *C. glabrata* (CG74) strains. Sabouraud dextrose agar (SDA) (HIMEDIA, Mumbai, India) was used for fungal growth. RPMI 1640 medium supplemented with sodium bicarbonate and glutamine (Thermo Fisher, Waltham, MA, USA), 2% dextrose (Merck, Darmstadt, Germany), 0.165 mol/L 3-(N-morpholino) propanesulfonic acid, 4-morpholine-propanesulfonic acid (MOPS) (Thermo Fisher, Waltham, MA, USA), and 0.2% chloramphenicol was used in the susceptibility tests. The commercial antifungals used in this study were fluconazole, ketoconazole, nystatin (Infinity Pharma, Campinas, Brazil), and amphotericin B (Cristália, São Paulo, Brazil). 

### 2.2. Synthesis of Silver Nanoparticles 

The synthesis of the AgNPs was performed following the procedure described by Ballottin et al. [11]. Briefly, the biobased synthesis consisted of two steps: (1) production of the fungal secreted protein extract, and (2) production of the AgNPs. In the first part, the fungus *Fusarium oxysporum* was grown on a solid culture medium consisting of 0.5% yeast extract and 2% agar and kept at 28 °C for 1 week. Then, approximately 1 cm^2^ of the solid medium containing the fungus was removed and transferred to a sterile liquid medium consisting of 0.5% yeast extract and 2% malt extract. The 500 mL culture medium was then incubated under 150 rpm at 28 °C for 6 days. The biomass obtained was then filtered using filter paper and washed with deionized water. Approximately 10 g of the biomass was resuspended in 100 mL of deionized water. This material was then stirred at 150 rpm at 28 °C for 72 h. The biomass showed a pinkish color and was filtered using a Buchner funnel and filter paper, and the filtrate was used for the synthesis of the silver nanoparticles. In the second step, the secreted protein extract, with a concentration of 0.1 g/mL, was mixed with 0.01 mol/L of AgNO3. The solution was maintained at 28 °C in flasks sealed with aluminum foil until the formation of the nanoparticles. The characterization of the *Fusarium oxysporum*-AgNPs was performed using UV-Vis, showing a characteristic maximum absorbance at 440 nm (Supplementary Figure S1), dynamic light scattering and transmission electron microscopy, as previously described [11,23] and showed in Table 1 and Supplementary Figure S2. The AgNP were spherical, showing a size of 28.0 ± 13.1 nm, a polydispersity of 0.231, and a zeta potential of −31.7 ± 2.8 mV (Figure S1) [11,23]. 

### 2.3. Fungal Samples and Ethical Aspects 

Clinical fungal isolates were obtained from patients screened by dentistry professionals at the Dentistry Ambulatory of the UNIME University, Salvador, Brazil. The samples from palate dentures presenting stomatitis lesions suggestive of oral candidiasis were collected using sterile swabs, inoculated on SDA supplemented with 0.2% chloramphenicol and incubated at 37 °C for 48 h [24]. The colonies were then isolated and maintained by weekly reinoculations. The Committee of Ethics on Research of the Institute of Health Sciences of the Federal University of Bahia approved this research under the protocol number 2.118.563, and an informed consent was obtained from all patients. 

The genomic DNA of the fungal isolates was obtained using the FastDNA Spin Kit (MP Biomedicals, Solon, OH, USA). Polymerase chain reactions (PCR) were performed using the primers ITS4 and ITS5 for the amplification of the complete internal transcribed spacer (ITS) region [25], and LROR and LR7 primers for the amplification of the fungal large subunit (LSU) rDNA. For the PCR, it was used 1.25 U DNA Polymerase, 50 mM MgCl2, 10 mM DNTPs, 10 pmol of each primer, 0.5% ultrapure DMSO, 0.75% BSA (stock at 1 μg/μL), 5 M betaine, and 50 ng DNA template, in a final volume of 50 μL. The thermocycling steps were: 2 min at 94 °C, 35 cycles of 1 min at 94 °C, 1 min at 55 °C, 1 min at 72 °C, and a final extension of 5 min at 72 °C. 

The purification of PCR products was carried out using an ethanol/EDTA (125 mM) precipitation protocol. The DNA sequencing was performed using the ABI 3730 automated sequencer (Applied Biosystems, Foster City, CA, USA). Consensus sequences were submitted to the Basic Local Alignment Search Tool (BLAST) for identification by similarity using the GenBank nucleotide sequences database of the National Center for Biotechnology Information (NCBI). For identification, similarities over 99% and an e-value equal to zero were considered. 

### 2.4. Broth Microdilution Assay

The fungistatic activities of the commercial antifungals and AgNPs were evaluated following the M27-A3 protocol from the Clinical Laboratory Standards Institute [26]. Briefly, the yeast strains were resuspended in sterile 0.9% saline solution and adjusted by spectrophotometry to a 600 nm optical density of 0.8–1.0, which corresponds to 0.5 at the McFarland scale. Subsequently, the yeast cells were diluted (1:50) in sterile saline solution and then diluted (1:20) in supplemented RPMI 1640 medium (Thermo Fisher, Waltham, MA, USA) to obtain a 2.5 × 10^3^ cells/mL inoculum. 

The AgNPs used in this study were serially diluted in sterile water for obtaining concentrations ranging from 1.95 to 1000 μg/mL. The commercial antifungal agents used as reference drugs were fluconazole (0.125–64 μg/mL), nystatin, ketoconazole and amphotericin B (0.0313–16 μg/mL) [26]. 

The inoculum (100 μL per well) was added to 96-well sterile culture plates. Subsequently, the same volumes of commercial fungicides and AgNPs at different concentrations were added to the wells. RPMI 1640 medium alone was used as a negative control and the inoculum without antifungal drugs was used as a positive control. The plates were incubated for 48 h at 37 °C and, after the incubation period, the wells were homogenized by pipetting, and the growth of *Candida* spp. was assessed by measuring the absorbance at 625 nm using a plate spectrophotometer (Multiskan FC Microplate Photometer, Thermo Fisher, Waltham, MA, USA). All the assays were performed in triplicate and were repeated three times. The minimum inhibitory concentration (MIC100) value, which represents the lowest concentration that inhibited 100% of the fungal growth, was determined as the minimum concentration of commercial antifungal agents and AgNPs that exhibited an OD reading similar to the negative control OD. For the determination of the minimum fungicide concentration (MFC100: minimal drug concentration able to kill 100% of the yeast cells), aliquots from each well of the broth microdilution assay were plated in SDA and then incubated at 37 °C for an additional 48 h.

According to the M27-S4 document [27], the breakpoint for fluconazole was considered for the classification of the fungal isolates according to the corresponding MIC100 (μg/mL), as follows: resistant (R) ≥ 8; dose-dependent susceptible (SDD) = 4; susceptible (S) ≤ 2, except for *C. glabrata*, where the interpretative endpoints were SDD ≤ 32 and R ≥ 64. Breakpoints for ketoconazole, nystatin and AmB were not available in M27-S4 document [23], and this classification was not performed for these drugs.

### 2.5. Biofilm Formation Interference Assay 

To evaluate the interference of AgNPs in biofilm formation, it was used the reference strains *C. parapsilosis* CP310 and *C. albicans* 2508. These species were chosen based on their AgNP susceptibility profile obtained for the planktonic cultures. 

The ability of the AgNPs to inhibit biofilm formation was evaluated as previously described [28]. The strains were incubated in SD Broth at 37 °C in a shaker at 250 rpm for 12–15 h. The cultures had their cell density adjusted to an OD600 of 0.38–0.5 with RPMI 1640 media and then added to a 96 well plate. The plates were incubated in a shaker at 250 rpm at 37 °C for 90 min. After that time, the media was aspirated, the wells were washed with sodium phosphate buffer (PBS 1×) pH 7.4, and AgNPs diluted in RPMI 1640 in concentrations ranging from 7.81 to 1000 μg/mL were added. 24 h later, the ODs were read in a spectrophotometer at 570 nm. The same controls that were reported above for the microdilution assay were used in this experiment. 

### 2.6. Scanning Electron Microscopy 

The scanning electron microscopy was performed as previously described [23], with minor modifications. Fungal suspensions cultured in the presence of half of the MIC100 concentration were deposited on 25 mm polytetrafluoroethylene (PTFE) membranes (0.45 μm pore size) (Millipore, Burlington, MA, USA) fixed with 2.5% glutaraldehyde in PBS pH 7.4 and washed twice with PBS. The membrane was soaked in 0.9% osmium tetroxide for 1 h and washed twice with distilled water. The dehydration was done by immersing the membranes in 30%, 50%, 70%, and 90% ethanol for 20 min, 20 min, 16 h, and 20 min, respectively, and then three times in 100% ethanol for 20 min each. The drying step was performed in a critical point dryer (Oerlikon Balzers, Balzers, Liechenstein), and the sputtering with gold was performed using a sputter coater (SCD-050, Leica, Wetzlar, Germany). Finally, the samples were analyzed using a scanning electronic microscope (JSM 5800LV, JEOL, Tokyo, Japan). 

### 2.7. Exogenous Ergosterol and Sorbitol Supplementation Assays 

The exogenous supplementation of ergosterol and sorbitol was conducted with the objective to determine the effect of AgNP treatment on the fungal surface. Sorbitol can prevent damages to the cell wall structure, whereas ergosterol protects the plasma membrane from the action of AgNPs. For the supplementation assays, the microdilution assay used for the drug susceptibility tests was performed as described above but with the addition of 0.8 M sorbitol or 200 μg/mL ergosterol in all samples and controls. This assay was conducted as previously described [29]. 

### 2.8. Statistical Analysis 

Dose-response curves were generated to assess the average response of the growth inhibition obtained in the broth microdilution tests. This analysis was performed with the GraphPad Prism 6.0 software (GraphPad Software, San Diego, CA, USA) using the nonlinear regression parameters for the dose-response inhibition (variable-slope) equation. The EC50 (concentration of drug capable of inhibiting 50% of yeast growth) was obtained through the dose-response inhibition (variable-slope) equation. The R square was greater than 0.65 for all the curves. The interference rates in biofilm formation assays were obtained in % using the following formula [30]:

[(OD_570_ of *Candida* spp. treated with AgNPs ∗ 100)/(OD570 of *Candida* spp. non-treated)] − 100

## 3. Results

### 3.1. Identification of the Candida Species Isolated from Denture Stomatitis Cases

The twenty *Candida* isolates from denture stomatitis lesions were identified through the sequencing of LSU and ITS gene fragments. The results showed the presence of three *Candida* species, with different frequencies (Table 2). *C. albicans* (14/20) was the most frequently isolated species, corresponding to 70.0% of the isolates, whereas *C. tropicalis* (5/20) and *C. dubliniensis* (1/20) were less abundant, with frequencies of 25.0% and 5.0%, respectively.

### 3.2. Susceptibility to Commercial Antifungal Drugs

The reference strains and the clinical isolates presented distinct susceptibility patterns for fluconazole and ketoconazole. All *C. albicans* reference strains were susceptible to fluconazole, with MIC_100_ ranging from 0.125 to 0.5 μg/mL. Of all *C. albicans* clinical isolates (14 in total), seven were susceptible (MIC_100_ ≤ 2 μg/mL), three were dose-dependent susceptible (S-DD) (MIC_100_ = 4 μg/mL), and four were resistant (MIC_100_ > 16 μg/mL) to fluconazole (Table 2). Thus, 28.57% of the *C. albicans* clinical isolates presented resistance to fluconazole. Regarding the MIC_100_ of fluconazole for the non-*Candida albicans* species, one *C. glabrata,* three *C. tropicalis*, and one *C. parapsilosis* strain were resistant (MIC_100_ ≥ 8 μg/mL).

The MFC_100_ of fluconazole for *C. albicans* isolates was markedly variable, with four *C. albicans* isolates presenting an MFC_100_ ranging from 2 to 16 μg/mL and ten isolates having non-determined MFC_100_. Considering the NCA species, the MFC_100_ for fluconazole presented by *C. dubliniensis* and three (of five) isolates of *C. tropicalis* was undetermined (Table 2).

Ketoconazole showed a fungistatic effect at low concentrations for all the *C. albicans* reference strains (14 in number, MIC_100_ 0.125 to 0.25 μg/mL) and clinical isolates (MIC_100_ 0.125 to 8 μg/mL), except for the PAC 12 isolate (undetermined MIC). The MFC_100_ was undetermined for ten *C. albicans* clinical isolates (MFC_100_ > 16 μg/mL). For eight *C. albicans*, four reference strains and four clinical isolates, the MFC ranged between 0.031 and 0.125 μg/mL. For C. *dubliniensis*, the MIC_100_ and MFC_100_ were 0.03125 and 0.125 μg/mL, respectively. Regarding the *C. tropicalis* clinical isolates, the MIC_100_ ranged between 0.03125 and 16 μg/mL, and the MFC could not be determined for three of the five isolates (Table 2). 

Nystatin and amphotericin B (AmB) presented the lowest variations in the MIC_100_ and MFC_100_ values for all *Candida* spp. tested in this study. The results obtained for nystatin showed that all *C. albicans* tested herein presented MIC_100_ values between 1 and 16 μg/mL and MFC_100_ ranging between 2 and 16 μg/mL, except for PAC 17, for which the MFC values could not be determined (>16 μg/mL). *C. dubliniensis* showed an MIC_100_ of 2 μg/mL and MFC_100_ of 4 μg/mL for nystatin. Regarding the response of *C. tropicalis* to nystatin, the MIC_100_ for the five isolates ranged between 0.5 and 4 μg/mL, and the MFC_100_ ranged between 2 and 8 μg/mL. Interestingly, AmB had the same MIC_100_ and MFC_100_ values for almost all strains (Table 2). The AmB MIC_100_ for *C. albicans* ranged between 0.25 and 4 μg/mL and the MFC ranged from 0.5 to 4 μg/mL. For *C. dubliniensis*, the MIC_100_ and the MFC_100_ values were the same (2 μg/mL). Finally, for *C. tropicalis*, the MIC_100_ of AmB ranged between 0.25 and 2 μg/mL and MFC_100_ ranged between 0.5 and 4 μg/mL. When each isolate was analyzed alone, the *C. albicans* PAC 17 clinical isolate presented the highest values of MIC_100_ and MFC_100_ among all the commercial drugs tested herein. Thus, this isolate can be considered as a potential multidrug resistant organism. 

### 3.3. Susceptibility to Silver Nanoparticles

The AgNPs presented a fungistatic effect at low concentrations for all the *Candida* spp. in this study. Fifteen (of 20) isolates from the oral cavity showed an MIC_100_ of 7.8 μg/mL, four isolates presented an MIC of 3.9 μg/mL, representing the lowest MIC_100_ determined herein, and *C. dubliniensis* showed the highest MIC_100_ (15.6 μg/mL) for AgNPs. The MFC_100_ values ranged from 15.6 to 500 μg/mL for the oral cavity isolates. For the reference strains, the AgNP had fungistatic activity with an MIC_100_ of 7.8 μg/mL for all the strains, except for *C. albicans* 2508 (MIC_100_ = 15.6 μg/mL). The MFC_100_ for the *C. parapsilosis* CP310 strain could not be determined (>1000 μg/mL). The MFC_100_ of the other reference strains ranged between 15.6 and 250 μg/mL (Table 2). 

**Figure 1:**
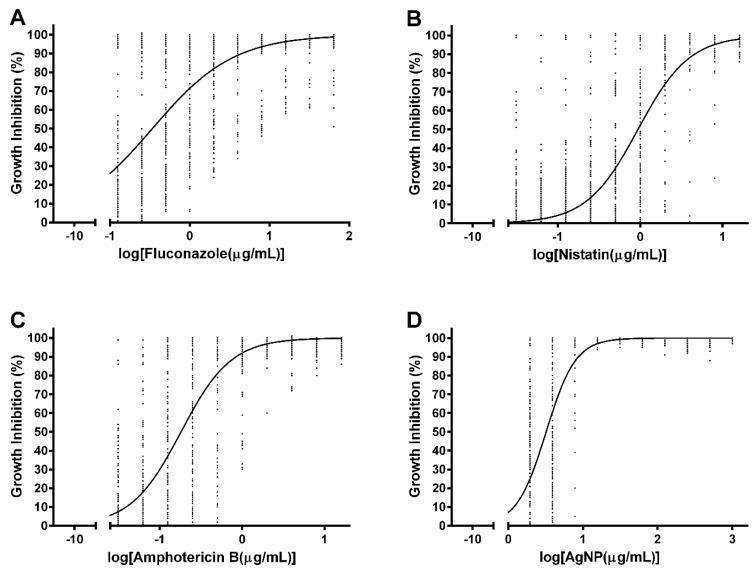
Dose-response curves of *Candida* spp. isolates treated with (**A**) fluconazole, (**B**) nystatin, (**C**) amphotericin B, and (**D**) AgNPs. For each concentration evaluated, 162 data points obtained for all *Candida* spp. were used to generate the nonlinear regression curves. EC_50_ values (in µg/mL) were 0.3389 (0.3096 to 0.3711) for fluconazole, 0.9402 (0.8815 to 1.003) for nystatin, 0.1810 (0.1710 to 0.1915) for amphotericin B, and 3.214 (3.109 to 3.322) for AgNPs. Statistical analysis and graphics were performed using the GraphPad Prism 6.0 software through nonlinear regression and dose-response inhibition tests.

The dose-response curves (Figure 1) show the response of all the *Candida* spp. specimens tested herein to fluconazole, nystatin, AmB and AgNPs. The analysis of the commercial fungicides showed that fluconazole had a high variance in the growth inhibition considering the same concentration and different isolates (Figure 1A). Nystatin and AmB presented a more stable per concentration response between isolates and *Candida* species (Figure 1B,C). Finally, the results showed a lower tolerance of all the *Candida* species to AgNPs, even at lower concentrations, presenting a similar behavior when considering the effective dose of AgNPs (Figure 1D).

### 3.4. Interference Effect of AgNPs in the Biofilm Formation

**Figure 2:**
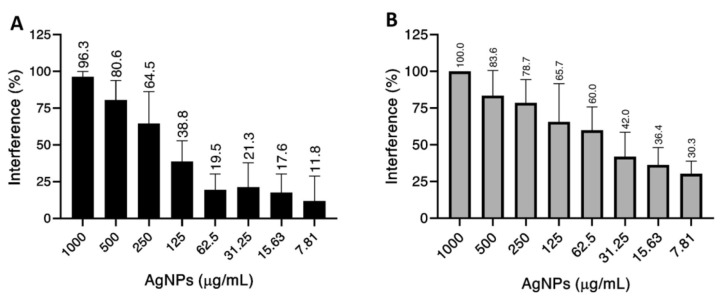
Interference of AgNPs in the biofilm formation by (**A**) *C. albicans* and (**B**) *C. parapsilosis*. The AgNPs concentrations ranged from 7.81 to 1000 mg/mL. The experiment was performed in quadruplicate. Percentages values of interference of AgNPs on biofilm formation are indicated above the columns. The results for *C. albicans* are indicated by black bars, while the results for *C. parapsilosis* are indicated by gray bars.

An interference on biofilm formation was observed in both *C. albicans* 2508 and *C. parapsilosis* CP310 reference strains when treated with the AgNPs (Figure 2). *C. albicans*, that was susceptible to the AgNPs in the planktonic form, showed 96.3% of interference in biofilm formation at the higher AgNP concentration tested herein (1000 µg/mL) (Figure 2A). This same concentration induced a 100% interference in *C. parapsilosis* biofilm formation (Figure 2B). The interference decreased to 80% in both species at 500 µg/mL of AgNPs. At lower concentrations (7.8–125 µg/mL), the AgNPs impaired biofilm formation to less than 40% in *C. albicans*, while for *C. parapsilosis* only concentrations below 31.25 µg/mL where able to reach this same inhibitory percentage.

### 3.5. Scanning Electron Microscopy of C. albicans and C. parapsilosis

**Figure 3:**
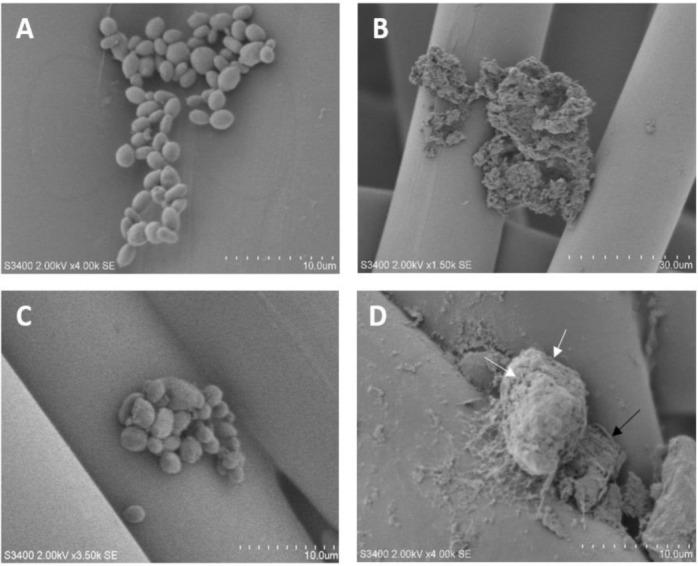
Scanning electron microscopy analysis of *Candida albicans* and *Candida parapsilosis* treated or not with AgNPs. (**A**) *C. albicans* 2508 and (**C**) *C. parapsilosis* CP310 cultured in the absence of AgNPs. (**B**) *C. albicans* 2508 and (**D**) *C. parapsilosis* CP310 treated with half of the AgNP MIC_100_. White arrows indicate pores in the cellular surface. The black arrow shows the residual biomass from other fungi lysed after treatment with the AgNPs. The scale bars are indicated in the right bottom of all the figures.

A scanning electron microscopy (SEM) analysis was performed to evaluate the effects of AgNPs on the fungi surface morphology. *C. albicans* 2508 and *C. parapsilosis* CP310 cultured without AgNPs showed a characteristic yeast morphology, varying from an ovoid to a spherical shape, occurring alone or grouped, and presenting an apparently intact cellular surface (Figure 3A,C). After incubation with the AgNPs, *C. albicans* appeared completely disrupted, showing cells debris without a defined morphology, as observed in AgNPs absence (Figure 3B). Agglomerates of biomass around the dried and disrupted *C. parapsilosis* cultured with AgNPs can be seen in Figure 3D. The SEM of *C. parapsilosis* after 48 h of treatment with AgNPs showed the presence of many pores on the cell surface. SEM analysis revealed that the AgNPs caused severe damage to the surface of the *Candida* spp.

### 3.6. Ergosterol and Sorbitol Supplementation

**Figure 4:**
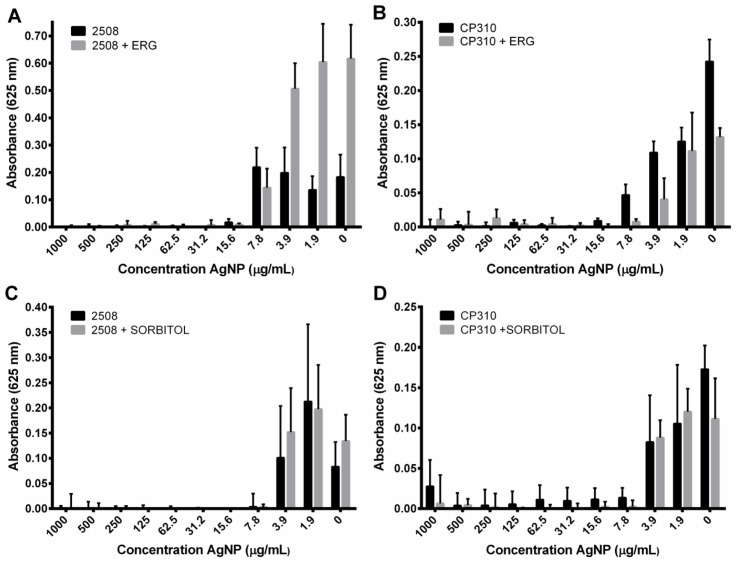
Ergosterol and sorbitol supplementation effects on the growth of *C. albicans* and *C. parapsilosis* isolates in the presence of different concentrations of AgNPs. Results obtained after ergosterol supplementation in (**A**) *C. albicans* 2508 and (**B**) *C. parapsilosis* CP310 cultures. Results obtained after sorbitol supplementation in (**C**) *C. albicans* 2508 and (**D**) *C. parapsilosis* CP310 cultures.

To evaluate if the surface damage caused by AgNPs can be prevented, *C. albicans* 2508 and *C. parapsilosis* CP310 were inoculated in SD media containing ergosterol (200 µg/mL) or sorbitol (0.8 M) supplementation, and were exposed to a range of AgNP concentrations. For *C. albicans* 2508 strain, ergosterol supplementation presented a protective effect at lower AgNP concentrations (Figure 4A,B). For *C. parapsilosis* CP310 strain, supplementation with ergosterol had a limited protection against AgNPs at the concentration of 1.9 µg/mL. Sorbitol supplementation presented no protective effect against AgNPs in the evaluated strains (Figure 4C,D). The results indicated that the damages in the fungal surface caused by AgNPs were primarily located in the plasmatic membrane. 

## 4. Discussion

*Candida* specimens are important etiologic agents of denture stomatitis [1]. The presence of *C. albicans* and NCA species in these infections has been already described [33]. In this study, the identification of the isolated *Candida* species showed that *C. albicans* was the most frequently isolated species, and *C. tropicalis* and *C. dubliniensis* were present in a lower abundance. Likewise, *C. albicans* and *C. tropicalis* were found causing chronic periodontitis at a similar frequency in Alagoas state, Brazil [34]. In Pará state, Brazil, *C. albicans* was the most frequent species associated with oral candidiasis, with a frequency of 78% [33]. In Spain, *C. albicans* accounts for 70% of *Candida* spp. isolated in oral candidiasis cases, followed by *C. glabrata* (8.6%), *C. parapsilosis* (7.4%), and *C. tropicalis* (3.3%) [31]. *C. dubliniensis* is a rare opportunistic fungus that causes oral cavity infections in patients with immunosuppression caused by HIV [32] and few descriptions of its prevalence in other fungal diseases are available [35].

Currently, four main classes of antifungal drugs, namely azoles, polyenes, allylamines, and echinocandins, are available for candidiasis treatment. Each class of these drugs has a specific action and a defined cellular target [36]. In this study, the analysis of four of the most used commercial antifungals showed that the majority of the *C. albicans* isolates were susceptible to fluconazole, whereas the NCA species were mostly SDD or resistant. However, in 71% of the *C. albicans* isolates, the MFC100 for fluconazole could not be determined. The resistance to fluconazole was detected in 36.8% of the *C. albicans* isolated from patients with chronic periodontitis from Alagoas State, in Brazil [34]. *C. parapsilosis* and *C. albicans* presented different susceptibility patterns to fluconazole, being the MIC higher for *C. parapsilosis* [37]. Fluconazole resistance is more common in NCA species than in *C. albicans* isolates [38]. The increase in the cases of *Candida* strains that are less susceptible or resistant to fluconazole indicates that the use of alternative drugs for the treatment of oral candidiasis is highly needed [39].

Nystatin and AmB are polyene antifungals; their mechanisms of action are associated with the content of ergosterol of the fungal membrane [40]. In the treatment of dental stomatitis, nystatin is an antifungal agent with high activity against *Candida* spp. isolates [31]. Unlike for azoles, the MIC100 and MFC100 for nystatin and AmB could be determined for all the isolates, except for one *C. albicans* isolate [40]. Regarding AmB, our results showed similar MIC100 and MFC100 values for almost all the isolates tested herein. A 71% agreement between the MIC and MFC values presented by *C. albicans* isolates treated with AmB has already been reported [41]. These results suggest that the same concentration of AmB can inhibit the growth and kill the fungi, a situation that can facilitate the disease treatment. 

There is a continuous evolution of drug resistance in *Candida* isolates [1]. Considering this situation, there is a constant demand for the development and discovery of new and safe broad-spectrum antifungal agents associated with a minimal toxicity to the host. In this context, AgNPs are nanotechnological compounds that present significant antimicrobial properties and low toxicity [42]. The AgNPs can be synthesized by different methods [11,17,43] and their antifungal [11,43,44,45] and antibacterial activities [43,46] have been correlated to their size, shape, and surface modifications [47,48]. It has been shown that the presence of the nicotinamide adenine dinucleotide (NADH) and NADH-dependent nitrate reductase enzymes are essential for the biosynthesis of AgNPs using microorganisms [49,50,51], and the reduction of silver ions occurs through the transfer of electrons from NADH by the reductase enzyme [49,50,51]. The biogenic AgNPs used in this study were synthesized using *F. oxysporum* secreted molecules. Considering the AgNPs used in dentistry, both prokaryotic and eukaryotic organisms can be used to synthesize AgNPs; however, plants are the most common organisms used in these AgNP syntheses [13]. The AgNP synthesized using *Aspergillus tubingensis* presented a positive zeta potential, spherical shape, and size of 35 ± 10 nm [44]. Among the different particle characteristics, the size and shape influence their antimicrobial properties; small particles (lower than 10 nm) that present a spherical shape are more effective against microorganisms [52]. In addition, the action of AgNPs appears to be highly related to the nanosize, which alters the level of silver ions released in the system and interferes with the surface energy [53]. 

Our results for the AgNP susceptibility assays using planktonic *Candida* showed that the AgNPs have a significant fungistatic effect at low concentrations, whereas the fungicidal effect of this nanoparticle showed more variable values, except for *C. parapsilosis* that presented an undetermined MFC. In opposite to our results, a previous study reported a strong activity of AgNPs against other *C. parapsilosis* isolate and it were also active against several *Candida* and bacterial species [43]. Radhakrishnan and collaborators [22] showed that citrated-reduced AgNPs inhibited *C. albicans* growth at lower concentrations and completely inhibit its growth at a concentration of 40 μg/mL.

The dose-response curves revealed that the inhibition of growth by AgNPs was significant, reaching 90% to 100% at low concentrations. These results are supported by the scientific literature, where other types of AgNPs have been reported to inhibit the growth of *C. albicans* at low concentrations (5 μg/mL) and caused a complete inhibition at high concentrations (40 μg/mL) [42]. In another study, biologically synthesized AgNPs, in combination or not with fluconazole, were effective against C. *albicans* [45]. The combination of AgNPs with simvastatin had a synergistic and additive effect against *Aspergillus* [54]. Thus, AgNPs can be used as an alternative or complementary treatment for fungal infections, considering their significant antimicrobial activities [42,45] and wound healing effects [46]. 

Biofilms are structured microorganism communities adhered to a surface and are considered as an antifungal resistance factor [4]. In our work, the AgNPs concentrations that were able to fully inhibit the biofilm formation by *C. albicans* and *C. parapsilosis* were 8-fold bigger than the MIC100 for both species. Recently, pure round AgNPs strongly inhibited formation and promoted disruption of *Candida auris* biofilm [55]. Additionally, the viability of fluconazole-resistant *C. tropicalis* biofilms was reduced when treated with AgNPs [56]. AgNPs embed into maxillofacial silicone elastomers reduced the *C. albicans* biofilm viability on this material [57]. All these studies showed that lower AgNPs concentrations were sufficient for a significant effect on *Candida spp.* biofilm. In addition, low doses can represent a decrease of unexpected effects on hosts [13]. 

The treatment with AgNPs induced a disruption of the fungal cells and the formation of pores on the cell surface, as shown by the SEM results. Similar analysis showed a high accumulation of nanoparticles outside the cells and the presence of small particles throughout the target cell cytoplasm [58]. Another study showed that the treatment of *Candida* spp. with other types of AgNPs disrupted the cell membrane and affected its integrity [59]. SEM and transmission electron microscopy analysis of *C. albicans* after treatment with AgNPs showed altered cellular morphology and ultrastructure [22]. Jalal et al. [43] showed that AgNPs are able to penetrate into *C. albicans,* leading to pore formation as a result of cell wall and membrane rupture. Additionally, *Candida* spp. treated with AgNPs can also exhibit alterations in the fluidity of the cell membrane and in the ergosterol content [22]. Furthermore, it has been suggested that the effects exerted by AgNPs on the fluidity of the membrane can be related to changes in the plasma membrane lipidic constitution and membrane depolarization [22,47]. 

Ergosterol is one of the most important constituents of the fungal cell membrane and plays a vital role in the stability of cells [60]. In the present study, the broth microdilution assays made with ergosterol supplementation resulted in a reduced susceptibility to AgNPs. The loss of sterols in the membrane leads to destabilization, resulting in an increased permeability and, thereby, enhanced sensitivity to drugs [60,61]. In addition, it was reported that the action of AgNPs also involves a reduction in the ergosterol content in the membrane [22]. Moreover, the importance of ergosterol is also based on the fact that its biosynthetic pathway is the target of most azoles, polyenes, and allylamines [61]. Sorbitol is considered an osmotic protector that can prevent damages to the cell wall [62]. Our results did not show any protection against the AgNPs action after sorbitol supplementation, besides the presence of pores evidenced by the SEM analysis. Additional investigation can lead to a better understanding of these mechanisms. However, literature evidence suggests that AgNPs can cause damage to fungal cells, acting against various cellular targets that can finally lead to cell lysis [17,22,43]. 

*Fusarium oxysporum*-based AgNPs showed significant antifungal activity on *Candida* spp. isolated from denture stomatitis, being able to impair the biofilm formation of *C. albicans* and *C. parapsilosis*. As a possible action mechanism, the plasmatic membrane can be a target of the nanoparticles. Thus, the biogenic AgNPs synthesized using *F. oxysporum* have a great potential as a complementary therapy of oral candidiasis.

## Figures and Tables

**Table 1:** Polydispersity (PDI) and zeta potential values of two batches of the silver nanoparticles (AgNPs).

Sample	PDI	Zeta Potential (mV)
AgNP Batch 1	0.22 ± 0.02	−29.8 ± 0.1
AgNP Batch 2	0.24 ± 0.04	−31.7 ± 2.8

**Table 2:** MIC_100_ and MFC_100_ values obtained for the *Candida* sp. reference stains and clinical isolates through broth microdilution assays using commercial fungicides and biogenic silver nanoparticles. All the tests were performed following the M27-A3 protocol from the CLSI [31]. MIC_100_ indicates the minimal inhibitory concentration and MFC indicates the minimal fungicide concentration. Breakpoints for fluconazole were obtained from the CLSI [32] M27-S4 document. S = Susceptible; SDD = Susceptible Dose-Dependent, R = Resistant.

Strain	Candida Species	Fluconazole (µg/mL)		Ketoconazole (µg/mL)		Nystatin (µg/mL)		Amphotericin B (µg/mL)		Silver Nanoparticles (µg/mL)	
		MIC_100_	MFC_100_	MIC_100_	MFC_100_	MIC_100_	MFC_100_	MIC_100_	MFC_100_	MIC_100_	MFC_100_
2508	*Candida albicans*	0.125 (S)	0.125	0.03125	0.03125	4	4	1	1	15.6	125
2517	*C. albicans*	0.25 (S)	0.125	0.03125	0.03125	4	8	1	1	7.8	250
3703	*C. albicans*	0.125 (S)	0.125	0.03125	0.03125	4	4	0.5	0.5	7.8	31.25
3704	*C. albicans*	0.5 (S)	0.25	0.03125	0.0625	2	4	0.5	0.5	7.8	62.5
PAC 06	*C. albicans*	0.25 (S)	2	0.0625	0.125	4	4	2	2	7.8	500
PAC 13	*C. albicans*	0.5 (S)	16	0.03125	>16	1	4	0.5	2	7.8	500
PAC 08	*C. albicans*	1 (S)	8	0.03125	0.125	2	4	2	2	7.8	250
PAC 18	*C. albicans*	1 (S)	>64	0.03125	>16	16	16	1	2	7.8	500
PAC 09	*C. albicans*	1 (S)	>64	0.0625	>16	2	4	0.5	0.5	7.8	500
PAC 10	*C. albicans*	1 (S)	>64	0.125	>16	2	4	0.5	0.5	7.8	250
PAC 03	*C. albicans*	1 (S)	>64	0.125	>16	2	2	0.25	0.5	7.8	62.5
PAC 19	*C. albicans*	4 (SDD)	16	0.125	1	8	8	0.5	0.5	7.8	500
PAC 20	*C. albicans*	4 (SDD)	>64	0.0625	>16	4	16	0.25	0.5	7.8	500
PAC 16	*C. albicans*	4 (SDD)	>64	0.125	>16	2	4	0.5	0.5	7.8	125
PAC 17	*C. albicans*	16 (R)	>64	0.03125	>16	16	>16	4	4	7.8	15.6
PAC 11	*C. albicans.*	>64 (R)	>64	0.0625	8	2	4	0.5	0.5	7.8	125
PAC 14	*C. albicans*	>64 (R)	>64	8	>16	4	4	0.5	0.5	3.9	15.6
PAC 12	*C. albicans*	>64 (R)	>64	>16	>16	2	2	0.5	0.5	7.8	31.25
CG 74	*Candida glabrata*	>64 (R)	>64	>16	>16	2	2	0.5	0.5	7.8	250
PAC 01	*C. dubliniensis*	32 (SDD)	>64	0.03125	0.125	2	4	2	2	15.6	500
CT 309	*Candida tropicalis*	>64 (R)	>64	8	>16	4	4	0.5	0.5	7.8	15.6
PAC 04	*C. tropicalis*	0.125 (S)	0.5	0.03125	0.03125	0.5	2	0.25	0.5	3.9	62.5
PAC 02	*C. tropicalis*	2 (S)	16	0.03125	0.125	2	4	2	2	7.8	125
PAC 15	*C. tropicalis*	2 (S)	>64	0.03125	>16	1	4	2	2	3.9	62.5
PAC 05	*C. tropicalis*	8 (R)	>64	0.25	>16	2	4	2	4	7.8	31.25
PAC 07	*C. tropicalis*	>64 (R)	>64	16	>16	4	8	1	1	3.9	15.6
CP 310	*Candida parapsilosis*	16 (R)	>64	0.125	>16	8	16	2	8	7.8	>1000

## Data Availability

The data presented in this study are available on request from the corresponding author.

## Supplementary Materials

The following supporting information can be downloaded at: https://www.mdpi.com/article/10.3390/nano12030501/s1, Figure S1: Transmission electron micrographs (TEM) of AgNPs, scales of 200 nm (left) and 50 nm (right). The silver nanoparticles obtained using *Fusarium oxysporum* (AgNPFU) showed a spherical-like shape, size 28.0 ± 13.1 nm, and were found to be stable for one year. It is possible to see the protein corona (light gray) around the Ag-core in the TEM image at the right. Figure S2: The absorption spectrum in UV-Vis of the synthesized AgNPS, with the characteristic surface plasmon resonance peak at 440 nm. The spectrum was measured using a UV-Vis HP8453 spectrophotometer using solutions placed in quartz cuvettes with a paht length of 10 mm. The spectra were taken in the range of 350 to 700 nm. The blank solution was prepared using the fungal filtrate by substituting the silver nitrate solution for distilled water.
