# Supplementary material for: Activity of Fusarium oxysporum-Based Silver Nanoparticles on Candida spp. Oral Isolates
Source: Nanomaterials (Basel). 2022 Jan 31;12(3):501. doi: 10.3390/nano12030501 (PMC8840154; doi:10.3390/nano12030501)
Supplement: Supplementary file 1 [file nanomaterials-12-00501-s001.zip › nanomaterials-1496216-supplementary.pdf]

nanomaterials

Supplementary Materials: Activity of *Fusarium oxysporum*-Based Silver Nanoparticles on *Candida* spp. Oral Isolates

Maísa Santos Fonseca [1], Daniela Méria Rodrigues [1], Ana Rita Sokolonski [2], Danijela Stanisic [3], Luiz Marcelo Tomé [4], Aristóteles Góes-Neto [4], Vasco Azevedo [5], Roberto Meyer [1], Danilo Barral Araújo [2], Ljubica Tasic [3] and Ricardo Dias Portela [1,*]

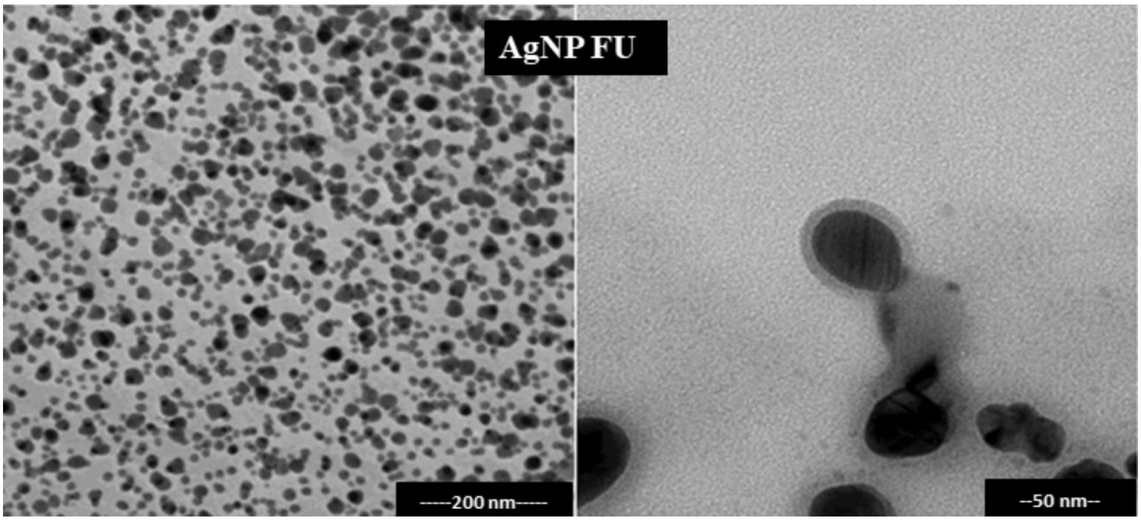

Figure S1. Transmission electron micrographs (TEM) of AgNPs, scales of 200 nm (left) and 50 nm (right). The silver nanoparticles obtained using *Fusarium oxysporum* (AgNPFU) showed a spherical-like shape, size 28.0 ± 13.1 nm, and were found to be stable for one year. It is possible to see the protein corona (light gray) around the Ag-core in the TEM image at the right.

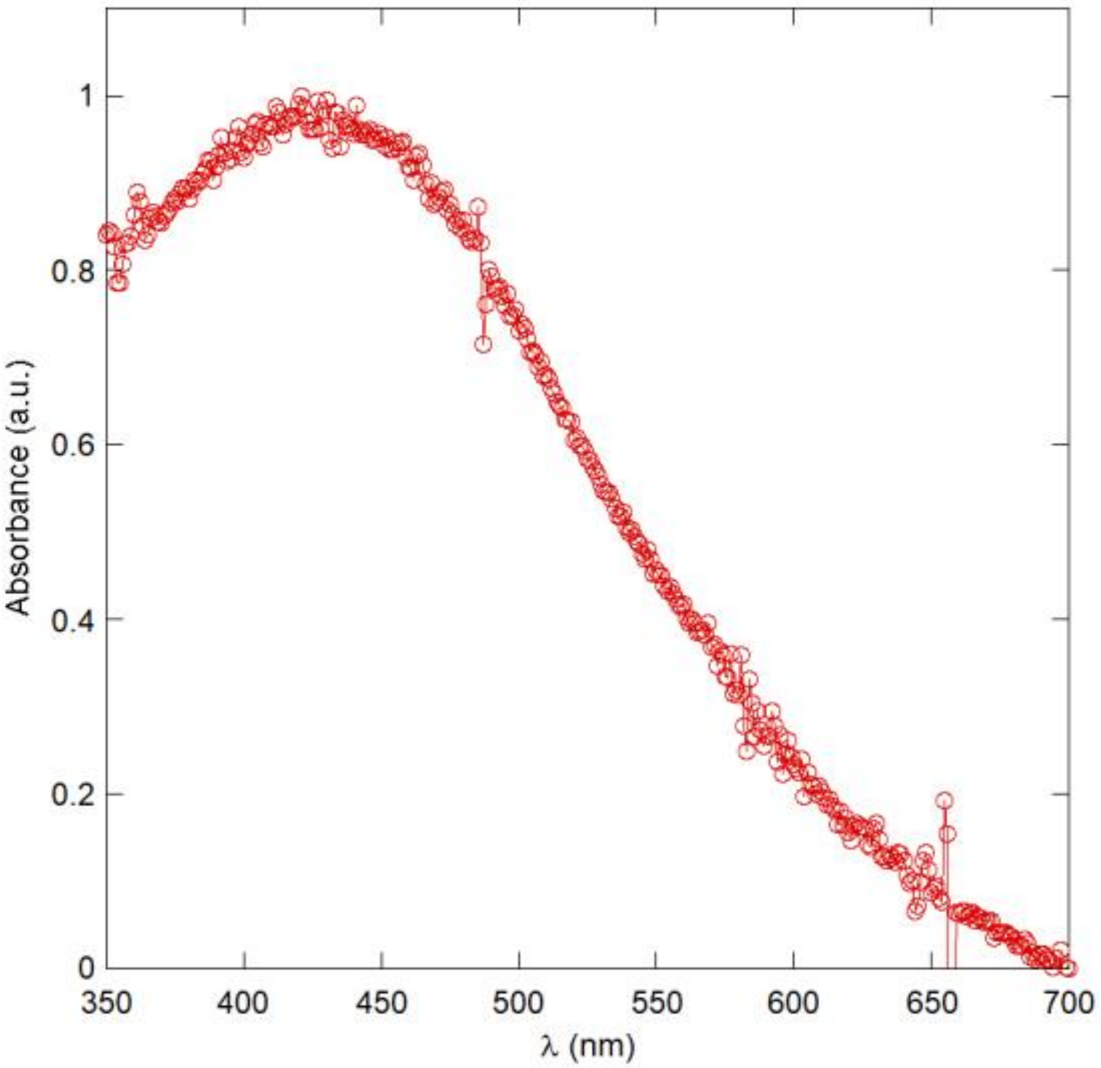

Figure S2. The absorption spectrum in UV-Vis of the synthesized AgNPS, with the characteristic surface plasmon resonance peak at 440 nm. The spectrum was measured using a UV-Vis HP8453 spectrophotometer using solutions placed in quartz cuvettes with a paht length of 10 mm. The spectra were taken in the range of 350 to 700 nm. The blank solution was prepared using the fungal filtrate by substituting the silver nitrate solution for distilled water.
